# Research hotspots and trends of skin barrier in atopic dermatitis in the past 24-year: a bibliometric analysis

**DOI:** 10.3389/fmed.2025.1539386

**Published:** 2025-03-12

**Authors:** Fangchang Huang, Xin Zhu, Xinglong Liang

**Affiliations:** ^1^The First Clinical College of Medicine, Guangdong Medical University, Zhanjiang, Guangdong, China; ^2^Department of Dermatology, Maoming People’s Hospital, Maoming, Guangdong, China

**Keywords:** atopic dermatitis, skin barrier, bibliometric analysis, VOSviewer, CiteSpace

## Abstract

**Background:**

Atopic dermatitis (AD) is a chronic, pruritic, inflammatory skin condition that imposes significant psychological and economic burdens on patients due to its recurring nature. Its etiology is multifactorial, involving interactions between genetic predispositions and environmental factors. The skin barrier serves as both a mechanical and immunological defense, and its structural damage and functional impairments significantly contribute to the pathogenesis of AD. This study aims to explore the future prospects and developmental trends of the skin barrier in the context of AD through a bibliometric analysis.

**Objective:**

To analyze the research status, hot spots and development trend of skin barrier in AD.

**Methods:**

Relevant studies were extracted from the Web of Science database and screened by researchers, with bibliometric analysis conducted using VOSviewer, CiteSpace, and other tools.

**Results:**

A total of 4,227 publications were identified over a 24-year research period. The United States is the leading contributor, with 1,263 publications, and demonstrates extensive collaboration with numerous countries. The journal with the highest number of publications is the Journal of Allergy and Clinical Immunology. The most prolific institutions is the University of California, San Francisco. Recent years have seen high citation intensity for keywords such as “dupilumab,” “barrier dysfunction,” and “gut microbiota”.

**Conclusion:**

The mechanism of the skin barrier in AD remains an area requiring ongoing research and analysis. Although significant progress has been achieved, future research will benefit from advancements in technology.

## 1 Introduction

Atopic dermatitis (AD) is a chronic and recurrent inflammatory skin disease with a high incidence. It is characterized by eczema-like changes, severe itching, and generalized dryness, frequently co-occurring with other atopic conditions like allergic rhinitis and asthma. AD prevalence reaches up to 20% in children and averages 2.1%–4.9% in adults (1, 2). Although the majority of AD patients with onset in childhood will gradually alleviate in adolescence or later, about 20%–30% of patients will continue to adulthood (3, 4), which can seriously affect the quality of life and mental health of patients (5), and increase the socio-economic burden (6).

The skin barrier consists of four interdependent layers: physical, chemical, microbial, and immune, collectively maintaining its structure and function (7). Increasing research indicates that disruption of the skin barrier is pivotal in AD pathogenesis. Structural and functional impairments contribute to inflammatory symptoms and infection susceptibility in AD patients (8).

Due to the burgeoning research interest in the link between skin barrier damage and AD pathogenesis, alongside the plethora of related publications, researchers face challenges in discerning the latest developments and research trends in this domain. Bibliometric analysis is a statistical method used to comprehensively review academic publications. It analyzes publications from specific periods, examining countries, institutions, journals, authors, keywords, and references to understand the knowledge structure and identify research frontiers or hotspots in a field (9). However, there has been no bibliometric review of studies focused on AD and the skin barrier.

Therefore, we retrieved literature data from the Web of Science (WOS) database^1^ to identify the most cited countries, authors, institutions, and journals in the AD and skin barrier field. This paper employs bibliometric analysis to summarize research on the skin barrier’s role in AD pathogenesis and treatment from 1999 to 2023, presenting current advancements, hotspots, and emerging trends. This aims to highlight pivotal research and guide new researchers toward future directions.

## 2 Methods

### 2.1 Data source and search strategy

WOS is one of the most widely accessed academic databases, encompassing over 12,000 high-quality journals and comprehensive citation records. Therefore, we have chosen WOS as our target database. We retrieved studies investigating the relationship between atopic dermatitis and the skin barrier from the WOS core literature database. The detailed search strategy was as follows: TOPIC = (atopic dermatitis) AND TOPIC = (skin barrier) AND DOCUMENT TYPES: (Article OR Review) AND LANGUAGE: (English).

Two researchers (FC Huang and X Zhu) independently screened the titles and abstracts, and downloaded the full text when necessary. The retrieval period was set from January 1, 1999, to December 31, 2023. We searched and collected research literature on atopic dermatitis and the skin barrier, including both research articles and reviews. Excluded from the analysis were news articles, conference papers, letters, duplicate publications, and literature unrelated to the topic under study. For articles meeting the inclusion criteria, we recorded all pertinent information, including title, author, institution, funding agency, geographic location, abstract, keywords, journal, year of publication, citation, and references.

### 2.2 Data analysis and visualization

Export the final documents obtained through screening in plain text format. Ensure to select “full records and cited references” for the export contents, and name the files using the format “download_*”. Utilize the bibliometric platform,^2^ VOSviewer (version 1.6.19), and CiteSpace (version 6.2.2) software to analyze and visualize the inputted data. The bibliometric platform primarily analyzes co-authorship and publications by countries/regions. VOSviewer is mainly used to analyze countries, institutions, authors, co-citations, and keyword co-occurrence, whereas CiteSpace is primarily used to analyze the burst of references and keywords. The world map depicting the number of published articles is visualized using Tableau 2024.1 software.

## 3 Results

**FIGURE 1:**
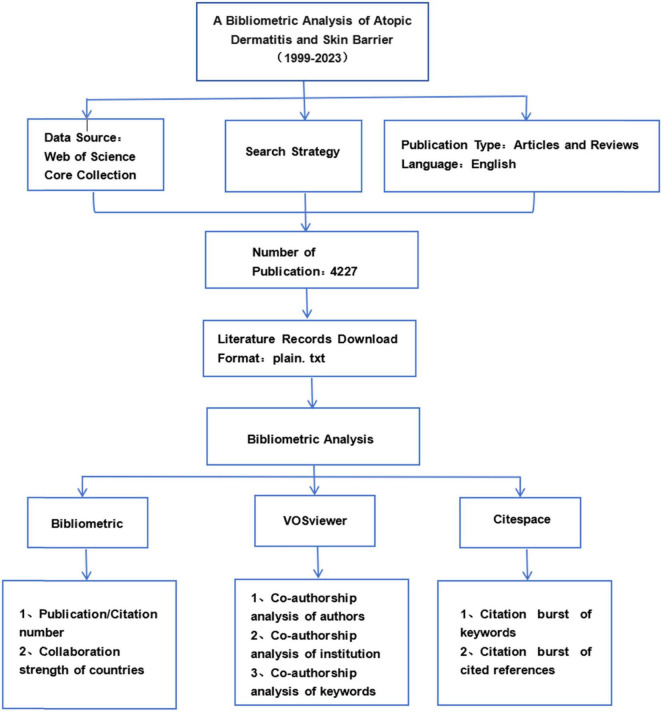
Flowchart of the searching stagey in the study.

In accordance with our search strategy, we retrieved 4,583 articles on AD and the skin barrier from the WOS database. Following the review process conducted by the researchers, a total of 4,227 articles were included in the present study. Based on the literature type, the 4,227 articles were categorized as 3,009 original articles and 1,218 reviews. Figure 1 presents the flowchart illustrating the methodology employed in this study.

### 3.1 Global tends in publications and citations

**FIGURE 2:**
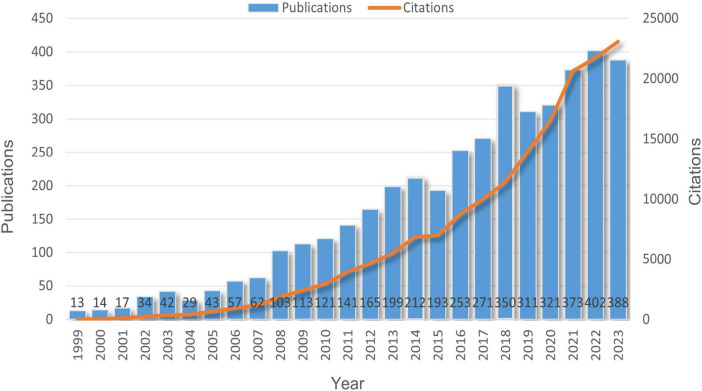
Global trend of publications and total citations on skin barrier in AD from 1999 to 2023.

All 4,227 articles were published between 1999 and 2023, covering a span of 24 years. Figure 2 illustrates the global trends in the annual number of published articles and their citations related to AD and the skin barrier. The data indicate a yearly increase in the number of studies on AD and the skin barrier since 1999, with the number of publications peaking at 402 in 2022. As of the search date, these papers have been cited a total of 164,853 times. Therefore, it can be concluded that the role of the skin barrier in the pathogenesis of AD remains a focal point of research.

### 3.2 Analysis of top productive countries/regions

**FIGURE 3:**
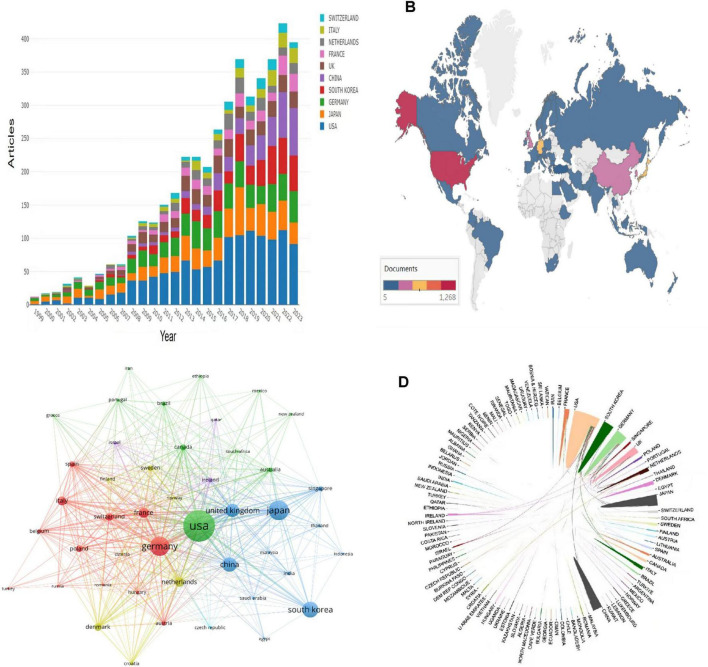
**(A)** Trends in the number of publications per year between 1999 and 2023 for the top 10 countries. **(B)** A world map based on the total number of publications in different countries/regions. **(C)** A collaborative network between major countries/regions generated using VOSviewer. **(D)** Country/area visualization maps for international cooperation.

Articles in this field were published across 92 countries. Figure 3A depicts the annual publication trends of the leading 10 countries over the last 24 years. As illustrated by the global map in Figure 3B, countries with higher numbers of published articles include the United States, Japan, Germany, and South Korea. According to Table 1, the United States ranks first with 1,263 articles, followed by Japan (627), Germany (602), and South Korea (445). Additionally, the United States leads in total citations (52,685), significantly ahead of Germany (37,658), which holds the second position. We utilized VOSviewer to analyze international cooperation among countries, as depicted in Figure 3C. With a minimum publication threshold set at 10, 45 countries were considered. Node size reflects publication counts, while lines between nodes indicate international cooperation, thicker lines denote stronger cooperation. However, when considering Figure 3D, it becomes evident that international cooperation remains relatively weak.

**TABLE 1:** Top 10 productive countries/regions related to the skin barrier in AD.

Rank	Country	Counts	Percentage	Citations	Average citation per paper
1	USA	1,268	30.00	72,429	57.12
2	Japan	627	14.83	25,584	40.80
3	Germany	602	14.24	37,658	62.55
4	South Korea	445	10.53	10,879	24.45
5	China	387	9.16	9,463	24.45
6	UK	350	8.28	23,838	68.11
7	France	235	5.56	12,239	52.08
8	Netherlands	207	4.90	12,219	59.03
9	Italy	181	4.28	6,431	35.53
10	Switzerland	154	3.64	11,369	73.82

### 3.3 Analysis on the output and influence of top journals

**FIGURE 4:**
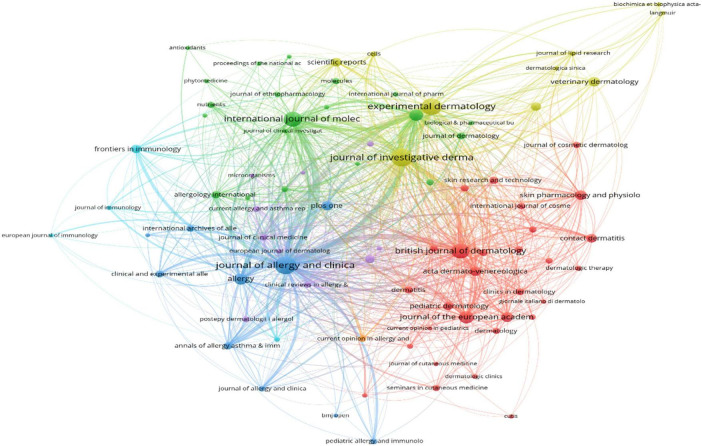
The relationship between journals.

The articles were published across 764 journals, with 84 journals publishing no less than 10 articles each. Table 2 lists the top 10 journals by publication volume and their latest impact factors. The three most prolific journals were *Journal of Allergy and Clinical Immunology* (194 articles, 4.59%), *JOURNAL OF INVESTIGATIVE DERMATOLOGY* (191 articles, 4.52%), and *Experimental Dermatology* (167 articles, 3.95%). Notably, *Journal of Allergy and Clinical Immunology* had a total citation count of 22,806, significantly higher than other journals. Among the top 10 journals listed in Table 2, five are based in the United States. Figure 4 illustrates the relationships between these journals.

**TABLE 2:** Top 10 Journals related to the research of skin barrier in AD.

Rank	Journal	Country	Counts	JCR (2023)	IF (2023)	Citations
1	Journal of Allergy and Clinical Immunology	USA	194	Q1	14.2	22,806
2	Journal of Investigative Dermatology	USA	191	Q2	6.5	14,512
3	Experimental Dermatology	Denmark	167	Q3	3.6	6,481
4	International Journal of Molecular Sciences	USA	138	Q2	5.6	3,519
5	British Journal of Dermatology	UK	134	Q1	10.3	5,798
6	Journal of The European Academy of Dermatology And Venereology	UK	93	Q2	9.2	3,816
7	Journal of Dermatological Science	Ireland	80	Q2	4.6	3,880
8	Allergy	UK	79	Q1	12.6	4,374
9	Journal of Drugs in Dermatology	USA	61	Q4	1.5	1,016
10	Plos One	USA	57	Q3	3.5	1,698

### 3.4 Analysis of the influence and cooperation of authors

**FIGURE 5:**
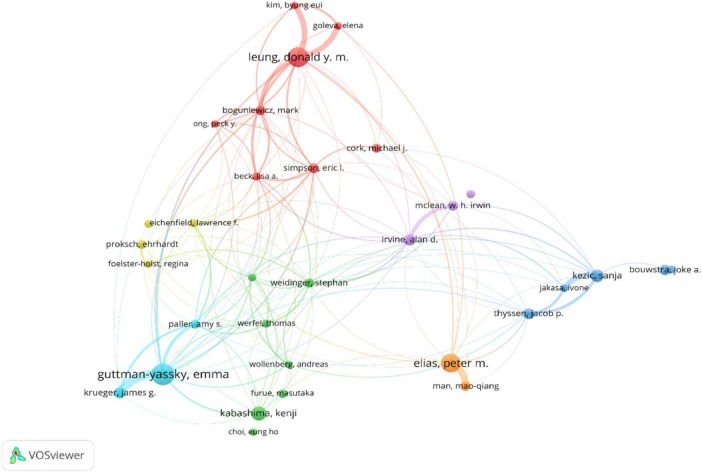
The visualization map of co-authorship analysis of authors.

A total of 16,923 authors have contributed to research on AD and skin barrier. Table 3 highlights the leading contributors: Guttman-Yassky, Emma, with 87 publications and an H-index of 86, followed by Leung, Donald Y. M. (77 publications, H-index of 129) and Elias, Peter M. (72 articles, H-index = 117), all three from the United States. Figure 5 shows the cluster analysis of cooperation analysis among researchers with more than 20 articles, revealing the cooperative relationship among researchers.

**TABLE 3:** The 10 most productive authors about skin barrier in AD.

Rank	Authors	Articles	Total citations	H-index	Institution and Country
1	Guttman-yassky, Emma	87	8700	86	Icahn School of Medicine at Mount Sinai, USA
2	Leung, Donald Y. M.	77	8251	129	National Jewish Health, USA
3	Elias, Peter M.	72	3874	117	US Department of Veterans Affairs, USA
4	Kabashima, Kenji	48	4164	74	Kyoto University, Japan
5	Kezic, Sanja	41	2158	45	University of Amsterdam, Netherlands
6	Irvine, Alan D.	36	5970	75	Trinity College Dublin, Ireland
7	Krueger, James G.	35	5032	114	Rockefeller University, USA
8	Marsella, Rosanna	33	808	23	University of Florida, USA
9	Thyssen, Jacob P.	33	1138	68	University of Copenhagen, Denmark
10	Bouwstra, Joke A.	32	2272	71	Leiden University, Netherlands

### 3.5 Analysis of institutional outputs and cooperation

**FIGURE 6:**
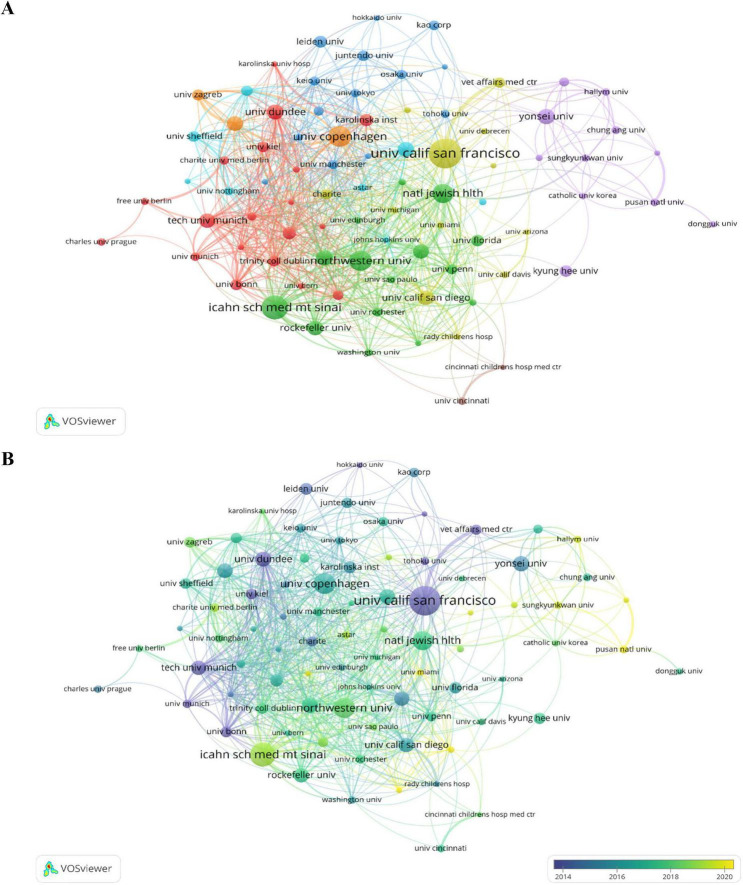
**(A)** The collaborative network of organizations. **(B)** The time-overlapping visualization for institutional co-authorship analysis.

Overall, there are 4,044 institutions have contributed to this research field, with Supplementary Table 1 detailing the top 10 institutions by article count. Figure 6A illustrates the collaborative networks among 84 institutions that have published more than 20 articles each. The University of California, San Francisco leads with 132 articles, indicative of earlier engagement in this area (Figure 6B). Following are the Icahn School of Medicine at Mount Sinai and the University of Copenhagen, with 104 and 89 articles, respectively. These institutions predominantly hail from Europe and the United States, although their distribution is markedly uneven, with a clear concentration at the top.

### 3.6 Research trends

#### 3.6.1 Citation frequency analysis and co-citation analysis

The most cited publications in a field reflect its research impact. Supplementary Table 2 lists the top 10 most cited publications, published between 1991 and 2012 (10–18). The highest cited article, “Common loss-of-function variants of the epidermal barrier protein filaggrin are a major predisposing factor for atopic dermatitis” by Palmer CAN, was published in 2006 (10). The second most cited article, “Atopic dermatitis,” appeared in the *NEW ENGLAND JOURNAL OF MEDICINE* in 2008 (11). Supplementary Figure 1A presents a visual co-citation network analysis of these references, and Supplementary Figure 1B highlights the top 25 references by citation volume. The blue line denotes citations from 1999 to 2023, and the red line shows the emergent citation range, with a minimum duration of 2 years. The pivotal literature remains Palmer CAN’s 2006 article. It can be seen from Supplementary Figure 1B that the citations in this field have exploded since 2006, and a large number of citations are still frequently cited, which indicates that the mechanism between AD and skin barrier is still a research hotspot in the next few years.

#### 3.6.2 Keyword co-occurrence and emergence analysis

Our study analyzed a total of 5,846 keywords. After applying a frequency threshold of 15 occurrences, we identified 114 keywords for inclusion. Using VOSviewer, we generated network visualization maps and superposition visualization maps of these keywords. Supplementary Figure 2A illustrates these maps: dot size indicates keyword frequency, line thickness reflects the strength of relationships, and color denotes keyword clustering. Keywords are grouped into three clusters: Cluster 1 (red) focuses on immune inflammatory factors in AD pathogenesis like “cell,” “cytokines,” “t cell,” “th2” and “mast cell,” etc. Cluster 2 (green) pertains to skin barrier definitions and mechanisms, including “dry skin,” “skin lipids,” “ceramides,” “epidermal differentiation,” “stratum corneum,” “tight junction,” etc. Cluster 3 (purple) centers on AD treatment, featuring terms like “biologics,” “dupilumab,” “emollients” and “topical corticosteroids.” Supplementary Figure 2B depicts the temporal overlap analysis network of these keywords, with purple and blue nodes indicating earlier appearances compared to green and yellow nodes, highlighting recent shifts in research focus. Keywords such as “th2”, “dupilumab”, and “Interleukin-13” have emerged prominently in recent years. Supplementary Figure 3 presents the top 25 keywords with the strongest citation bursts. Keywords like “dry skin” (1999–2011), “human stratum corneum” (2002–2014), “barrier dysfunction” (ongoing since 2020), “gut microbiota” (2020–2023), and “probiotics” (2021–2023) continue to attract significant attention, indicating that these keywords represent the research hotspots in recent years and even in the future.

## 4 Discussion

This paper presents a comprehensive bibliometric study on the research related to the skin barrier in AD pathogenesis, using visualization tools such as VOSviewer and CiteSpace to thoroughly analyze the existing literature. It is the first study to summarize both historical research findings and the current state of knowledge on the skin barrier in AD, while also identifying development trends and future research hotspots related to skin barrier in AD through the use of these widely adopted bibliometric tools.

Analysis of 4,227 articles from the WOS database reveals a gradual increase in studies on the correlation between the onset of AD and the skin barrier since 1999, with the United States ranking first in terms of the number of published articles and influence, having published 1,268 articles. Additionally, half of the top 10 journals, publishing institutions, and authors are based in the United States, reflecting its dominance, which is closely linked to its significant economic resources and investment in medical research. Furthermore, Figure 3A shows a gradual increase in publications from China, South Korea, and other countries in recent years, indicating growing international interest in the study of the pathogenesis of the skin barrier in AD. It is anticipated that more countries and researchers will engage in this field of study in the future. Consequently, scientific advancement is closely tied to robust national economic support and international collaboration.

Impact factor, JCR and total citations are effective indicators for assessing the influence of journals. Notably, the *Journal of Allergy and Clinical Immunology* and the *Journal of Investigative Dermatology* have the highest number of publications, often taking on the responsibility of disseminating significant research in their respective fields. As shown in Table 2, all 10 of the leading journals are from developed countries in Europe and the United States, likely reflecting the influence of their advanced economic development. Despite notable contributions from Japan, South Korea, and China, Asian countries have not yet ranked first in terms of publication volume. This indicates that Japan and other Asian countries possess the potential to establish journals with significant international influence in the future.

As early as 1985, Werner and Lindberg utilized an evaporometer to measure water loss from the stratum corneum of both dry and clinically normal skin in patients with AD. The results showed that patients with AD exhibited a higher rate of water loss from the stratum corneum, particularly in regions of dry skin. This suggests a potential defect in skin barrier function among patients with AD. Additionally, studies have demonstrated that the water-binding capacity of the stratum corneum in AD patients is diminished, and alterations in the lipid composition of the skin surface may contribute to increased water loss. Consequently, alterations in the composition and structure of the stratum corneum may impair skin barrier function in patients with AD (19). The number of citations of a document serves as an indicator of its influence. In the most cited literature, “Common loss-of-function variants of the epidermal barrier protein filaggrin are a major predisposing factor for atopic dermatitis,” Palmer et al. identified two gene mutations (R510X and 2282del4) significantly associated with AD through genotyping both affected individuals and healthy controls, leading to the identification of filaggrin loss-of-function alleles. Filaggrin, as a crucial protein, facilitates the terminal differentiation of the epidermis and the formation of the skin barrier; thus, its loss of function represents a significant pathogenic factor in AD. These mutations are present in over 9% of the European population (10). This study reveals the critical role of impaired skin barrier function in the development of AD and further investigates the genetic mechanisms and pathogenesis of AD.

Keywords often represent the core content of research, and word frequency can indicate shifts in research hotspots. As illustrated in Supplementary Figures 2B, 3 topics such as “th2,” “mast cell,” “interleukin-13,” “gut microbiota” and “probiotics” have garnered significant attention from researchers in recent years. The pathogenesis of AD, as explored through its diagnosis and treatment, involves genetic susceptibility, epidermal barrier disruption, local and systemic immune imbalances, and interactions with environmental factors (20). Notably, the immune imbalance is primarily characterized by Th1/Th2 imbalance mediated by Th2-type immunity (21). Wang et al., conducted a bibliometric analysis of 2,168 studies related to mast cells (MCs) and AD, concluding that Th2 cytokines, such as IL-4 and IL-13, released by MCs exacerbate the immune imbalance in AD and further promote inflammation. IL-13, IL-4, NFKB1, BGF-1, and CD4 are key genes associated with MCs and AD. These genes primarily regulate inflammatory responses, leukocyte activation, cytokine production, and cell activation. The development of biologics and small-molecule drugs targeting MCs represents a crucial direction for the future treatment of AD (22). Gut microbiota influences the development of AD by modulating immune function, maintaining skin barrier integrity, and regulating neuroendocrine signaling. Probiotics and prebiotics have demonstrated potential therapeutic effects in regulating gut microbiota and restoring intestinal microecological balance. Studies suggest that probiotics can alleviate pruritus in AD patients by modulating gut microbiota, indicating potential therapeutic efficacy in improving AD symptoms (23, 24). Additionally, dysregulation of the skin microbiome plays a significant role in the pathogenesis and progression of AD. The increased colonization of *Staphylococcus aureus* on the skin of AD patients leads to a reduction in skin bacterial diversity and induces skin inflammation through the release of inflammatory mediators such as IL-1α and IL-24β, which are closely linked to the onset and severity of AD (16, 25, 26). Symbiotic microbes, such as *Staphylococcus epidermidis*, help maintain skin barrier integrity by secreting ceramides (27). This suggests that future studies will further investigate the complex relationship between the microbiome and AD, potentially providing new therapeutic strategies (28). We anticipate that the diagnosis and treatment of AD will become more diverse in the future.

AD remains a major contributor to the global burden of skin-related diseases. However, research resources are predominantly concentrated in a few developed countries, and international cooperation remains limited. Despite evidence showing a rising prevalence of AD in developing countries, many patients continue to receive ineffective or inappropriate treatments (29). In the current global information age, countries are able to communicate effectively. Developed nations should leverage their advantages to enhance international cooperation and collectively advance research on AD.

## 5 Limitation

It is important to acknowledge that this study has certain limitations. Firstly, this study analyzed literature exclusively from the WOS database. The literature collection is not sufficiently comprehensive, potentially leading to biased results. Future research should expand the search database to achieve a more comprehensive analysis. Secondly, the citation threshold applied to the screened articles may have excluded recent high-quality papers that have not yet reached the ideal number of citations, leading to potential research bias.

## 6 Conclusion

In summary, the role of the skin barrier in the pathogenesis of AD has been extensively investigated, and significant progress has been achieved. However, further advancements are still necessary. Developing countries should actively seek and maintain close collaboration with developed nations, such as the United States, Japan, and Germany. Additionally, we anticipate that future research hotspots will focus on the following areas: (1) Targeted factors in inflammatory pathways; (2) The role of gut microbiota in AD and related treatments; 3. Long-term management and comorbidities associated with AD. This bibliometric study facilitates a more accurate and in-depth investigation of the skin barrier in AD.

## Data Availability

The original contributions presented in this study are included in this article/Supplementary material, further inquiries can be directed to the corresponding author.

## Footnotes

1
https://www.webofscience.com/wos/

2
https://bibliometric.com/
