# Supplementary material for: Research hotspots and trends of skin barrier in atopic dermatitis in the past 24-year: a bibliometric analysis
Source: Front Med (Lausanne). 2025 Mar 12;12:1539386. doi: 10.3389/fmed.2025.1539386 (PMC11936790; doi:10.3389/fmed.2025.1539386)
Supplement: Supplementary file 1 [file Supplementary_file_1.docx]

Supplementary Material

Supplementary Figures and Table

1. Supplementary Figures

**Figure S1.** The analysis of co-citation cited reference and strongest citation bursts.

**Figure S2.** The co-occurrence network of keywords.

**Figure S3.** Top 25 keywords with robust citation burst.

| **A** | 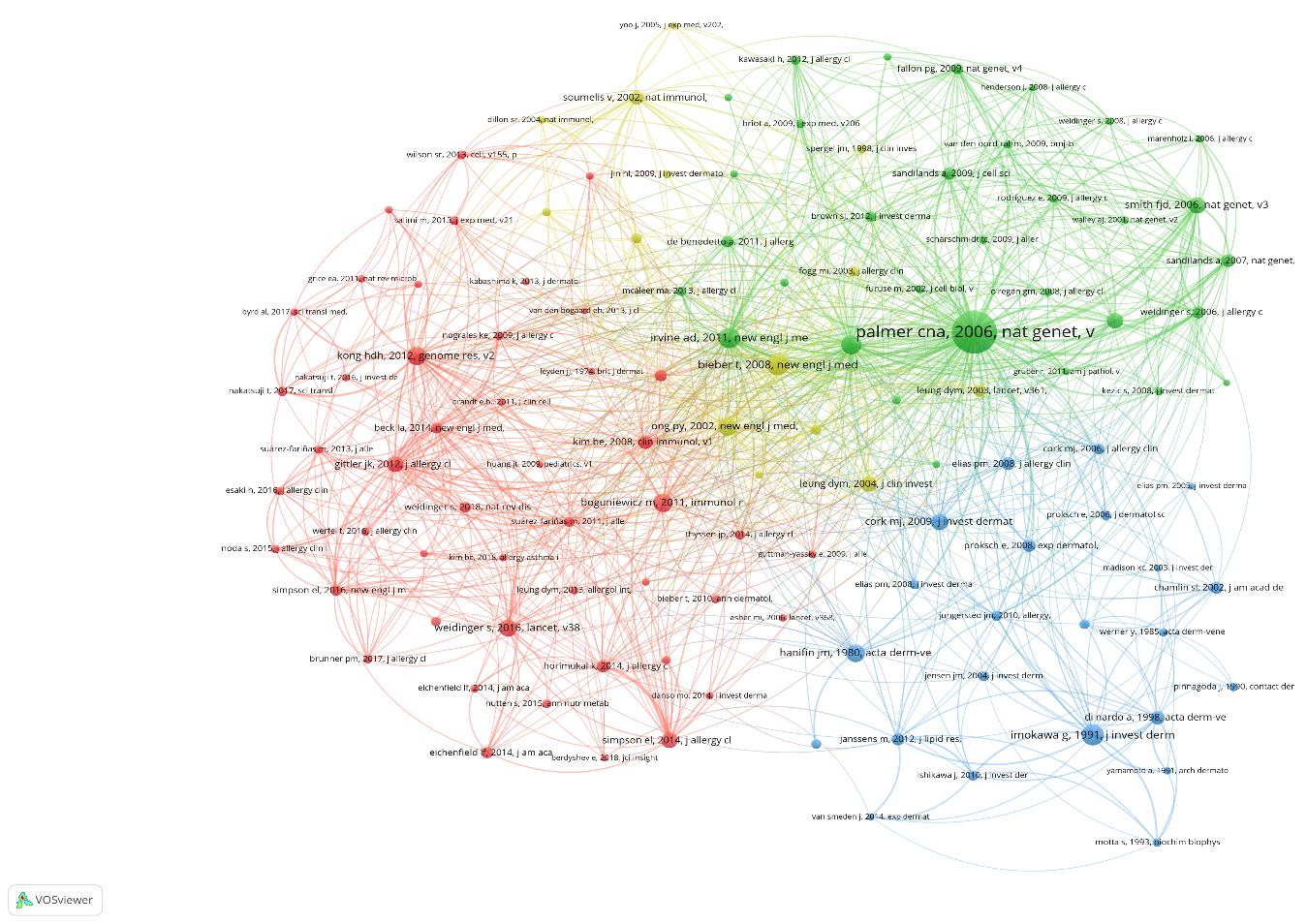 |
| --- | --- |
| **B** | 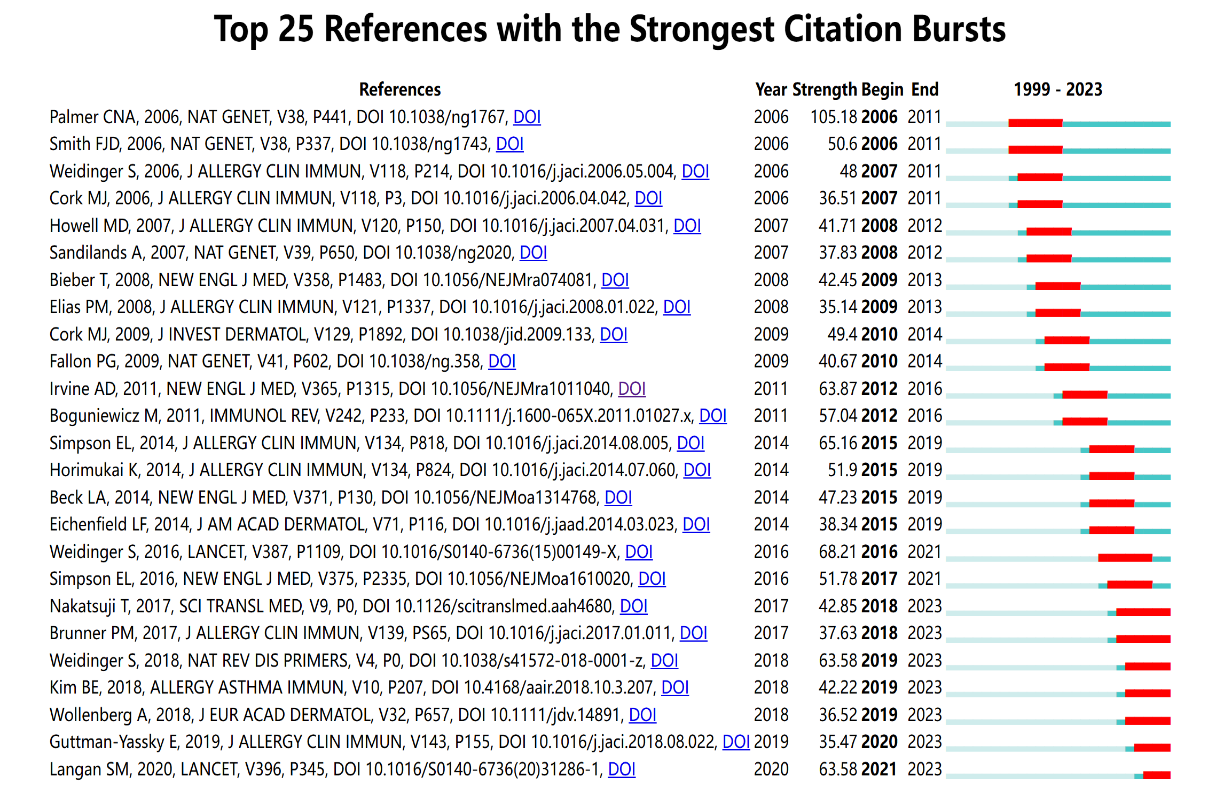 |
|  | |

**Figure S1. (A)** The analysis of co-citation cited reference.**(B)** The visualization map of top 25 references with the strongest citation bursts from 1999 to 2023.

| **A** | 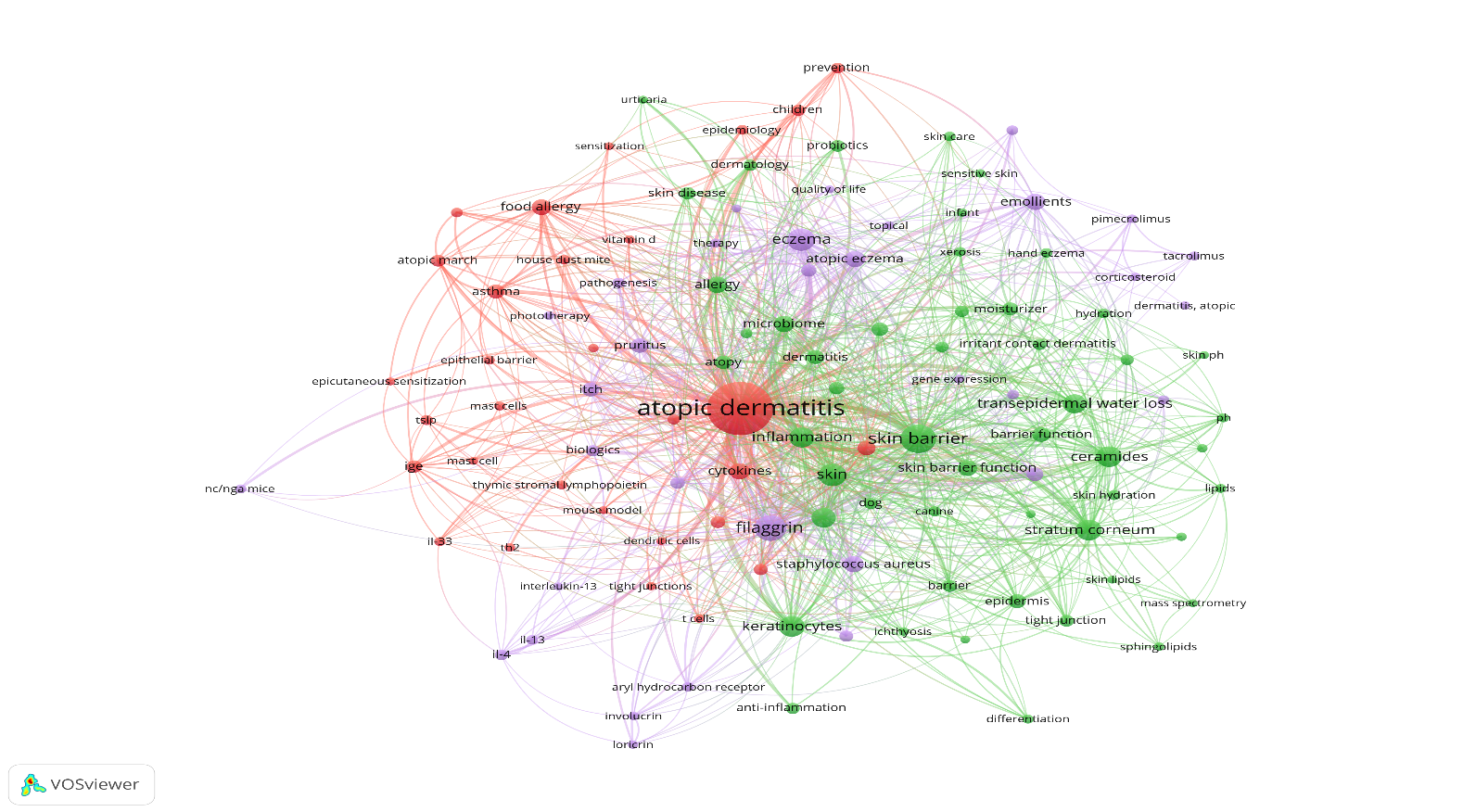 |
| --- | --- |
| **B** | 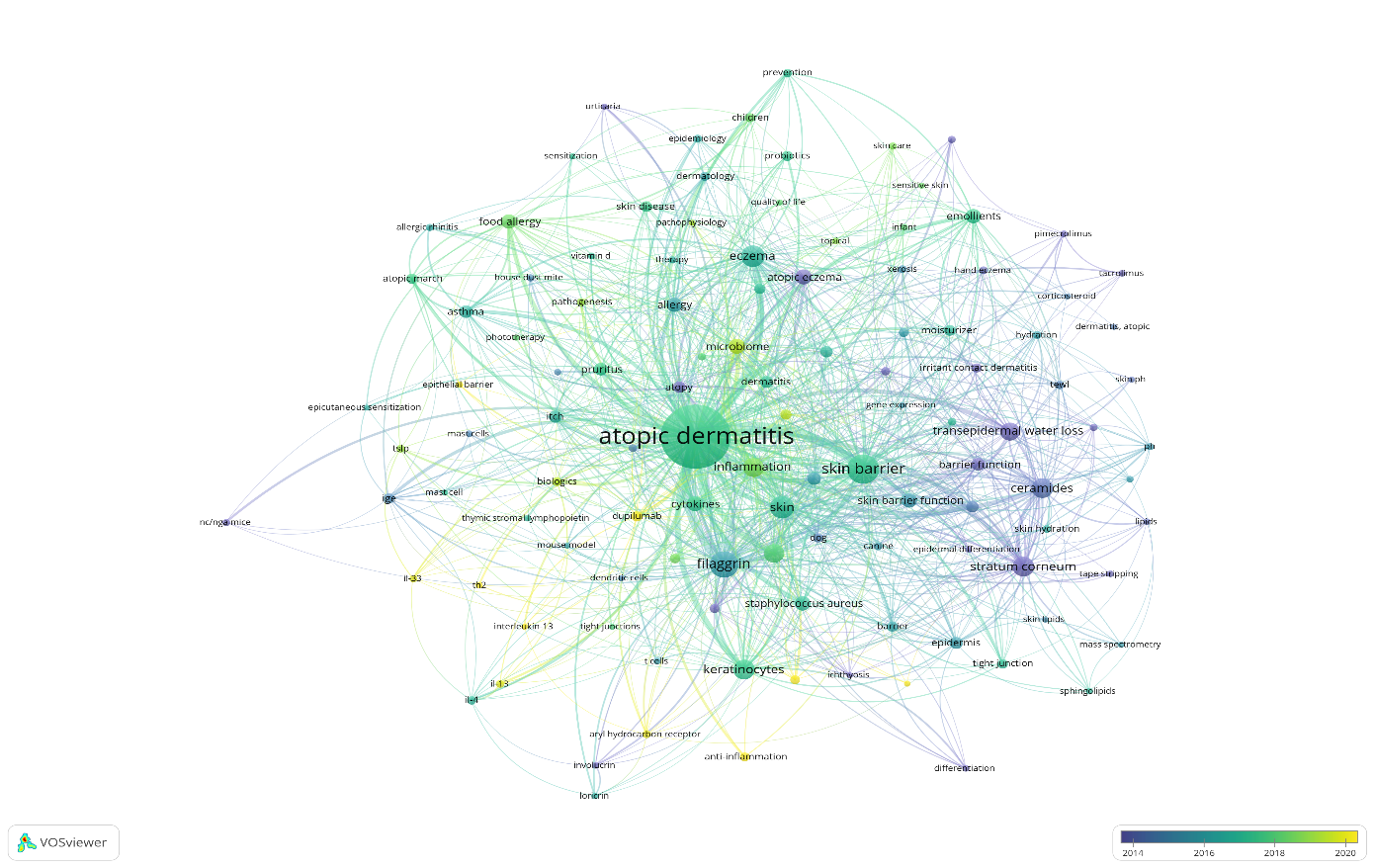 |

**Figure S2. (A)** The co-occurrence network of keywords. **(B)** The overlay visualization map of keywords.

| 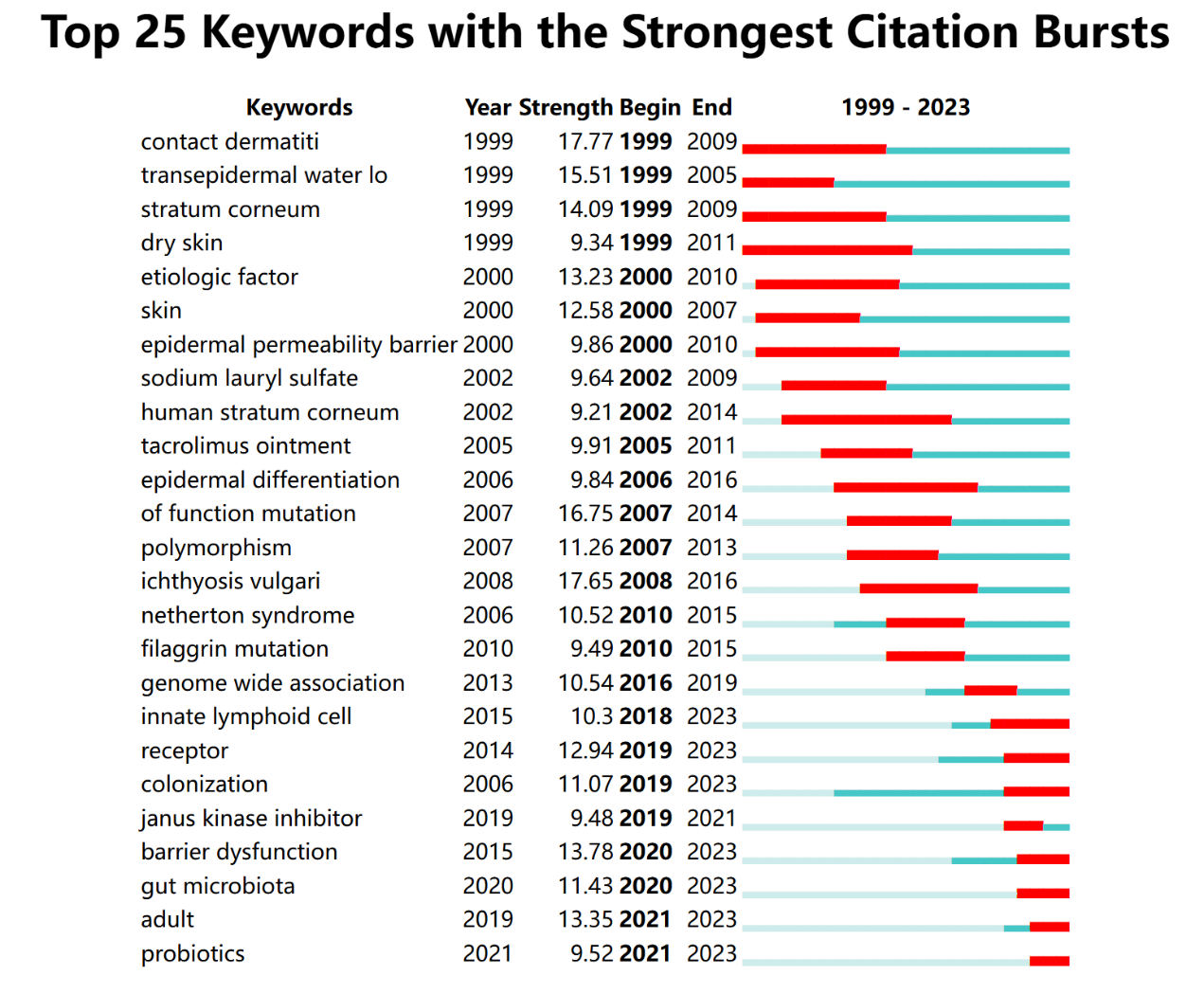 |
| --- |

**Figure S3.**Top 25 keywords with robust citation burst.

1. Supplementary Table

**Table S1.** Top 10 institutes the publications concerning of skin barrier in AD.

**Table S2.**Top 10 co-cited references concerning the research of skin barrier in AD.

**Table S1.** Top 10 institutes the publications concerning of skin barrier in AD.

| **Rank** | **Institutions** | **Country/Regions** | **Articles** | **Total citations** |
| --- | --- | --- | --- | --- |
| 1 | University of California, San Francisco | USA | 132 | 8051 |
| 2 | Icahn School of Medicine at Mount Sinai | USA | 104 | 7919 |
| 3 | **University of Copenhagen** | Denmark | 89 | 2977 |
| 4 | Northwestern University | USA | 88 | 5197 |
| 5 | National Jewish Health | USA | 81 | 7041 |
| 6 | Oregon Health and Science University | USA | 64 | 3413 |
| 7 | Yonsei University | Korea | 64 | 2015 |
| 8 | University of Dundee | UK | 63 | 10074 |
| 9 | Kyoto University | Japan | 61 | 4444 |
| 10 | Universiteit van Amsterdam | Netherlands | 61 | 3013 |

**Table S2.**Top 10 co-cited references concerning the research of skin barrier in AD.

| **Title** | **Journals** | **Author** | | **Year** | | **Citations** |
| --- | --- | --- | --- | --- | --- | --- |
| Common loss-of-function variants of the epidermal barrier protein filaggrin are a major predisposing factor for atopic dermatitis(10) | NATURE GENETICS | Palmer CAN, etc. | | 2006 | | 843 |
| Atopic dermatitis(11) | NEW ENGLAND JOURNAL OF MEDICINE | | Bieber T, etc. | | 2008 | 370 |
| Decreased level of ceramides in stratum corneum of atopic dermatitis: an etiologic factor in atopic dry skin?(12) | Journal of Investigative Dermatology | | [IMOKAWA, G](https://webofscience.clarivate.cn/wos/author/record/9353985),etc. | | 1991 | 366 |
| Differential factors associated with challenge-proven food allergy phenotypes in a population cohort of infants: a latent class analysis(13) | NEW ENGLAND JOURNAL OF MEDICINE | | [Irvine, AD](https://webofscience.clarivate.cn/wos/author/record/1100555), etc. | | 2011 | 349 |
| Cytokine modulation of atopic dermatitis filaggrin skin expression(14) | JOURNAL OF ALLERGY AND CLINICAL IMMUNOLOGY | | [Howell, MD](https://webofscience.clarivate.cn/wos/author/record/912822), etc. | | 2007 | 321 |
| Atopic dermatitis: a disease of altered skin barrier and immune dysregulation(15) | Immunological Reviews | | [Boguniewicz, M](https://webofscience.clarivate.cn/wos/author/record/19262607), etc. | | 2011 | 315 |
| Temporal shifts in the skin microbiome associated with disease flares and treatment in children with atopic dermatitis(16) | Genome Research | | [Kong, HDH](https://webofscience.clarivate.cn/wos/author/record/393327), etc | | 2012 | 306 |
| Endogenous antimicrobial peptides and skin infections in atopic dermatitis(17) | NEW ENGLAND JOURNAL OF MEDICINE | | [Ong, PY](https://webofscience.clarivate.cn/wos/author/record/1806575),etc. | | 2002 | 298 |

**Table 5.***Cont.*

| DIAGNOSTIC FEATURES OF ATOPIC-DERMATITIS | ACTA DERMATO-VENEREOLOGICA | [HANIFIN, JM](https://webofscience.clarivate.cn/wos/author/record/8730428), etc. | 1980 | 294 |
| --- | --- | --- | --- | --- |
| Atopic dermatitis(18) | LANCET | [Weidinger, S](https://webofscience.clarivate.cn/wos/author/record/111309), etc. | 2016 | 285 |
